# The Catalog of Microbial Genes and Metagenome-Assembled Genomes from the Gut Microbiomes of Five Typical Crow Species on the Qinghai–Tibetan Plateau

**DOI:** 10.3390/microorganisms12102033

**Published:** 2024-10-08

**Authors:** Boyu Tang, You Wang, Yonggang Dong, Quanchao Cui, Zhanhao Zeng, Shunfu He, Wenxin Zhao, Zhuoma Lancuo, Shaobin Li, Wen Wang

**Affiliations:** 1 State Key Laboratory of Plateau Ecology and Agriculture, Qinghai University, Xining 810016, China; tby1213@163.com (B.T.); wy09122289@163.com (Y.W.); dyg0516@126.com (Y.D.); cqc5614@163.com (Q.C.); 2 College of Eco-Environmental Engineering, Qinghai University, Xining 810016, China; 3 Animal Disease Prevention and Control Center of Qinghai Province, Xining 810016, China; zzh790911@163.com; 4 Xining Wildlife Park of Qinghai Province, Xining 810016, China; heshunfu_xnzoo@126.com (S.H.); 15297013980@163.com (W.Z.); 5 College of Finance and Economics, Qinghai University, Xining 810016, China; lczm1980@163.com; 6 College of Life Sciences, Yangtze University, Jingzhou 434025, China

**Keywords:** *Corvidae*, avian microbiome, gut microbiota, metagenome-assembled genomes, gene catalog

## Abstract

While considerable progress has been made in understanding the complex relationships between gut microbiomes and their hosts, especially in mammals and humans, the functions of these microbial communities in avian species remain largely unexplored. This gap in knowledge is particularly notable, given the critical roles gut microbiomes are known to play in facilitating crucial physiological functions, such as digestion, nutrient absorption, and immune system development. Corvidae birds are omnivorous and widely distributed across various habitats, exhibiting strong adaptability and often displaying the traits of accompanying humans. However, to date, information on species composition, sequenced genomes, and functional characteristics of crow gut microbes is lacking. Herein, we constructed the first relatively comprehensive crows gut microbial gene catalog (2.74 million genes) and 195 high-quality and medium-quality metagenome-assembled genomes using 53 metagenomic samples from five typical crow species (*Pyrrhocorax pyrrhocorax*, *Corvus dauuricus*, *Corvus frugilegus*, *Corvus macrorhynchos*, and *Corvus corax*) on the Qinghai–Tibetan Plateau. The species composition of gut microbiota at the phylum and genus levels was revealed for these five crow species. Simultaneously, numerous types of prevalent pathogenic bacteria were identified, indicating the potential of these crows to transmit diseases within the local community. At the functional level, we annotated a total of 356 KEGG functional pathways, six CAZyme categories, and 3607 virulence factor genes in the gut microbiomes of the crows. The gut microbiota of five distinct crow species underwent a comparative analysis, which uncovered significant differences in their composition, diversity, and functional structures. Over 36% of MAGs showed no overlap with existing databases, suggesting they might represent new species. Consequently, these findings enriched the dataset of microbial genomes associated with crows’ digestive systems. Overall, this study offers crucial baseline information regarding the gut microbial gene catalog and genomes in crows, potentially aiding microbiome-based research, as well as an evaluation of the health risks to humans from the bacterial pathogens transmitted by wild birds.

## 1. Introduction

Birds are one of the most diverse groups of vertebrates in the world, with more than 10,000 species (https://datazone.birdlife.org/species/taxonomy, accessed on 1 August 2024), and they occupy virtually every corner of the globe. They play a crucial role in various ecosystems due to their long flight distances, extensive movements, and complex diets. Consequently, the gastrointestinal tracts of birds harbor a diverse community of microbes, which have been increasingly found to play a crucial role in multiple aspects of the host’s health [1]. These gut microbes are not only vital for nutrition by aiding in the digestion and absorption of various nutrients [2] but also modulate the host immune system, helping to maintain immune balance and resilience [3]. Furthermore, these microbes provide protection against pathogenic microorganisms by competitively excluding them and producing antimicrobial compounds [4]. Additionally, they contribute to increasing the host’s tolerance to various stressors, such as dietary changes or environmental stress, by promoting metabolic flexibility and enhancing the overall resilience of the bird [5]. It is becoming evident that these gut microbiomes in wild birds are not only diverse and crucial but also heavily influenced by various environmental factors [6]. Birds serve as exemplary model species for examining the interactions between hosts and the gut microbiota, given their intricate life histories, varied dietary preferences, distinct mating behaviors, and capabilities for long-distance migration [7,8,9]. Moreover, these characteristics likely make their gut microbiota unique in comparison to other vertebrate classes [10].

The study of the avian gut microbiome has significantly expanded, driven primarily by a heightened interest in bird populations and the rapid progress in high-throughput sequencing technologies [11,12]. However, our comprehension of the variety and role of gut microbes is mainly centered on bird species that are either economically valuable [13] or rare and endangered conservation birds [14,15]. Research investigating the gut microbiome of wild birds, especially those that extensively overlap with human activity areas, is significantly scarce. Most bird species live remotely from human settlements, while those coexisting with humans show gut microbiota more deeply impacted by human activities [16]. Expanding our understanding of the gut microbiome in wild birds that coexist with humans will significantly aid in discerning the interplay between wild avian species and human interactions.

The Corvidae family consists of approximately 131 species (HBW and BirdLife Taxonomic Checklist v8.1, https://datazone.birdlife.org/species/taxonomy, accessed on 1 August 2024) and includes both small and large-sized passerines found globally, from temperate to tropical regions. These birds have demonstrated remarkable adaptability, thriving in a variety of environments, being particularly adaptive to urban habitats and living in proximity to humans [17]. Omnivorous diet, advanced cognition [18], and reduced predation and persecution pressures in urban areas are essential factors promoting corvids’ living in urban settings. Consequently, corvid species are frequently characterized as urban adaptors and sometimes even as exploiters, making them suitable candidates for studying the impacts of urbanization on birds [19]. Corvids in urban areas have both positive and negative impacts on the environment, providing ecosystem services such as seed dispersal and early detection of contaminants while also causing disservices through waste foraging, noise production, and disease transmission [20]. Research on Corvidae has increasingly focused on understanding their role in transmitting pathogens, particularly due to the high population densities of urban areas. For instance, infections with multiple *Sarcocystis* species were common among corvids, indicating that these birds may play a significant role in the transmission dynamics of these parasites [21]. Chang et al. employed metatranscriptomic methods to identify candidate viral pathogens associated with birds in Corvidae [22]. The ability of Corvidae birds to thrive in human-dominated environments and their role in disease transmission make them not only important from a conservation standpoint but also from a public health perspective.

To bridge the gap in our understanding of gut microbiota in wild Corvidae birds, we performed a thorough metagenomic analysis of five prevalent Corvidae species on the Qinghai–Tibetan Plateau, including Red-billed choughs (*Pyrrhocorax pyrrhocorax*), Daurian jackdaws (*Corvus dauuricus*), Rooks (*Corvus frugilegus*), Large-billed crows (*Corvus macrorhynchos*), and Northern ravens (*Corvus corax*). Among these species, only the gut microbiota of Red-billed choughs was studied by our research group using 16S rDNA sequencing [23]. As for the metagenomic data of these species, there are none at all. Our work will provide fundamental gut microbial gene and genome collections for these five common crow species on the Qinghai–Tibetan Plateau, and will help in understanding the natural variation in wild birds’ microbiomes, as well as how they differ in congeneric wild bird species that live in proximity to human settlements.

## 2. Materials and Methods

### 2.1. Ethics Statement

This study was conducted in accordance with the guidelines for the care and use of experimental animals established by the Ministry of Science and Technology of the People’s Republic of China (Approval number: 2006-398). The research protocol was reviewed and approved by the Ethical Committee at Qinghai University.

### 2.2. Sample Collection

**Figure 1:**
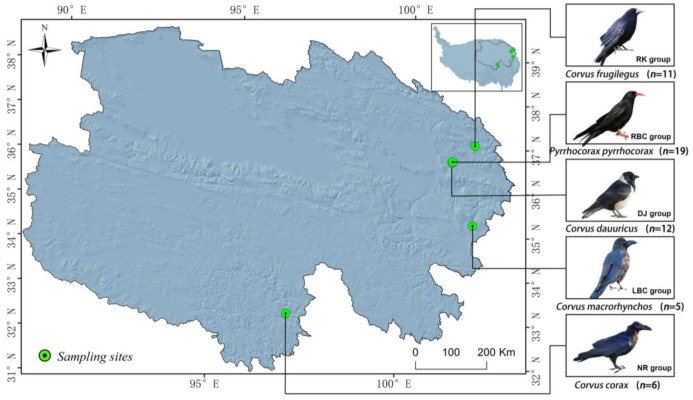
Map of avian sampling locations.

In this study, three species of crows were captured from a local area within the Qinghai Province of China (Figure 1). These crows included Red-billed choughs (*Pyrrhocorax pyrrhocorax*), Daurian jackdaws (*Corvus dauuricus*), and Rooks (*Corvus frugilegus*). A total of 42 individuals were captured and classified into three groups: Red-billed choughs (RBC group, *n* = 19), Daurian jackdaws (DJ group, *n* = 12), and Rooks (RK group, *n* = 11). After transporting the individuals to the laboratory, dissections were performed. The intestines were extracted, and the intestinal contents were squeezed out. All intestinal contents were stored in a freezer at −80 °C until further use. Additionally, fresh fecal samples were collected from two other crow species: Large-billed crows (*Corvus macrorhynchos*) (labeled as the LBC group, *n* = 5), and Northern ravens (*Corvus corax*) (labeled as the NR group, *n* = 6) (Figure 1). These samples were obtained directly after defecation, in the immediate vicinity of our sampling staff, who then promptly froze them using liquid nitrogen. The samples were subsequently transported to the laboratory and stored at −80 °C for future analysis.

### 2.3. Metagenomic Sequencing

Genomic DNA was isolated from all 53 samples utilizing the Qiagen QIAamp DNA Stool Mini Kit (Qiagen, Germany), in accordance with the supplier’s guidelines. To eliminate RNA contamination, the extracts were treated with DNase-free RNase. DNA concentrations were measured using the Qubit 2.0 fluorimeter (Invitrogen, Waltham, MA, USA). The purity of the DNA was determined with a Nanodrop (Thermo Scientific, Waltham, MA, USA) by assessing the 260/280 and 260/230 absorbance ratios. Metagenomic sequencing libraries were created and sequenced by Shanghai Biozeron Biological Technology Co., Ltd. (Shanghai, China). Briefly, a Paired-end (PE) library with an insert size of 450 bp was created for each sample. This was followed by high-throughput sequencing on the Illumina NovaSeq 6000 platform (Illumina, SD, USA), generating PE reads of 2 × 150 bp in length.

In this study, all bioinformatics software and tools were used, with default settings, unless specifically mentioned. We ensured the high quality of our reads by excluding those of low quality, which included reads with uncertain “N” bases, adapters, and any contamination from host DNA (*Corvus macrorhynchos* reference genome DRR250114, https://trace.ncbi.nlm.nih.gov/Traces/?view=run_browser&acc=DRR250114&display=metadata, accessed on 1 September 2023). This process used Trimmomatic (v.0.39) [24] and Bowtie2 (v.2.4.1) [25] as part of the KneadData pipeline (https://github.com/biobakery/kneaddata, accessed on 1 September 2023). The assembly process of a single sample was executed utilizing the MEGAHIT (v.1.2.9) [26]. During the procedure, an optional setting was applied, namely the “-k-list 21, 29, 39, 49, 59, 69, 79, 89, 99, 109, 119, 129, 141”. This particular parameter facilitates the exploration of varying k-mer sizes to refine the assembly’s effectiveness. Contigs that were assembled and exceeded 500 base pairs in length were subsequently subjected to gene prediction analysis using Prodigal (v.2.6) [27]. We developed a comprehensive and non-redundant catalog of microbial genes through the process of clustering the predicted genes. This was achieved using CD-HIT, with a sequence identity threshold set above 95% (v.4.8.1) [28]. The longest sequence in each cluster represented the unique gene set. The high-quality reads from each sample were aligned against the unique gene set using BWA-MEM (v.0.7.17), and abundance profiles of genes (alignment length ≥ 50 bp and sequence identity > 95%) were calculated in transcripts per million (TPM), with corrections for variations in gene length and mapped reads per sample. Taxonomic information at all levels (phylum, class, order, family, genus, and species) was obtained using Diamond (v.0.9.22) to align unigenes against the NCBI NR database, with parameters set as e-values ≤ 1 × 10^−5^ and scores ≥ 60 [29]. The taxonomic profile served as the foundation for computing alpha diversity, which was employed to assess the species richness and overall diversity present within the samples. The Bray–Curtis distance was calculated and visualized using Principal Coordinate Analysis (PCoA) for β-diversity. The process of functional annotation involved the alignment of amino acid sequences, which were translated from the predicted genes, with the KEGG [30], the CAZy database (http://www.cazy.org/ accessed on 1 November 2023), the VFDB database (http://www.mgc.ac.cn/VFs/, accessed on 1 November 2023), and PHI-base (http://www.phi-base.org/index.jsp, accessed on 1 December 2023) using DIAMOND (v.0.9.22), with e-values ≤ 1 × 10^−5^.

### 2.4. Genome Binning

The process of metagenomic binning on the single-sample assembly was executed utilizing MetaBAT2 (v.2.12.1) [31] with default parameters. The completeness and contamination of all bins were assessed using CheckM (v.1.1.1) [32]. Bins with >50% completeness and <10% contamination were classified as high quality MAGs. The resulting MAGs were processed through a dereplication step utilizing dRep (v.3.4.2) [33]. This procedure was performed with a default threshold of 99% average nucleotide identity (ANI). The ANI between genomes was calculated by FastANI [34]. Each bin was taxonomically classified using the Genome Taxonomy Database (GTDB-Tk) (v.2.3.0) [35]. We utilized the CoverM tool, specifically version 0.6.1, available at https://github.com/wwood/CoverM (accessed on 1 February 2024), to align the cleansed reads against the high-quality bins. This alignment was essential for computing the Transcripts Per Million (TPM) for each bin within the metagenomes. Subsequently, the genes within the MAGs were identified and then translated into their respective amino acid sequences using Prodigal software (v.2.6) [27].

### 2.5. Statistical Analysis

All statistical analyses were conducted utilizing R software version 4.4.1. For making statistical comparisons, we employed nonparametric methods. Specifically, the Wilcoxon test was used when comparing two groups, while the Kruskal–Wallis test was applied for comparisons involving multiple groups. The multiple test correction was conducted using Benjamini–Hochberg false discovery rate. Biomarkers that exhibited significant differences between groups were determined using the linear discriminant analysis effect size (LEfSe) [36]. For all statistical tests, a *p* value of less than 0.05 was considered statistically significant.

### 2.6. Data Availability

The raw sequence data from this paper have been deposited in the Genome Sequence Archive at the National Genomics Data Center, China National Center for Bioinformation/Beijing Institute of Genomics, the Chinese Academy of Sciences (GSA: CRA018740, CRA018741, CRA018833, CRA018712), accessible at https://ngdc.cncb.ac.cn/gsa (accessed on 1 September 2024).

## 3. Results

### 3.1. Metagenome Sequencing Data

To systematically compare and analyze the gut microbiomes of common corvid species on the Qinghai Plateau, China, we collected a total of 53 samples from five representative crow species, which included 42 intestinal content samples from three crow species: *Pyrrhocorax pyrrhocorax* (the RBC group, *n* = 19), *Corvus dauuricus* (the DJ group, *n* = 12), *Corvus frugilegus* (the RK group, *n* = 11), and 11 fecal samples from two additional crow species: *Corvus macrorhynchos* (the LBC group, *n* = 5) and *Corvus corax* (the NR group, *n* = 6). In total, metagenomic sequencing generated 9,371,335,632 raw paired-end metagenome reads (mean = 176,817,653.4, maximum = 317,031,310, and minimum = 117,146,210) (Table S1). An average of 26.52 Gbp were obtained per sample (Table S1). A total of 9,188,238,572 clean reads (mean = 173,362,991.9, maximum = 314,322,984, and minimum = 113,350,564) were obtained after filtering out the low-quality reads (Table S1). The draft genome sequence of *Corvus macrorhynchos* (NCBI accession number: DRP006609) was used to remove any potential host sequence contamination in the samples, ultimately yielding a total of 1,054,813,572 high-quality sequences for subsequent analysis (Table S1).

### 3.2. Microbial Composition and Variation in the Gut Microbiome at the Phylum Level

**Figure 2:**
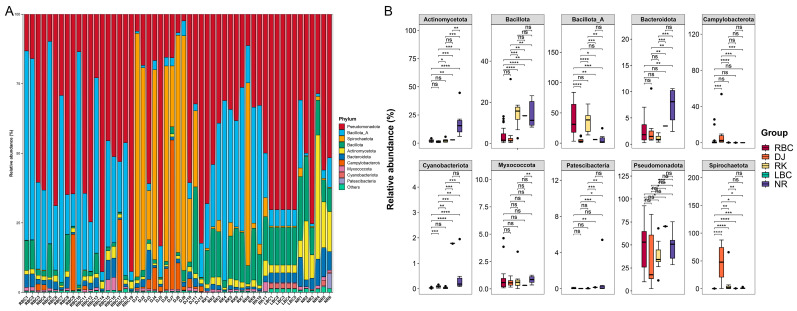
Microbial composition at phylum level. (**A**) Stacked bar chart of relative abundance of the top 10 phyla in each sample. (**B**) Comparison of the top 10 phyla among different groups. Statistically significant differences are indicated as follows: ns, *p* > 0.05; * *p* < 0.05; ** *p* < 0.01; *** *p* < 0.001; **** *p* < 0.0001.

The relative abundance of the three taxonomic ranks, including phylum, genus, and species, in each sample, as well as the differential analysis among various groups, were analyzed. A total of 132 bacterial phyla were detected among the five groups (Table S2). At the phylum level, the overall community profile constituted dominantly Pseudomonadota (54.83%), Bacillota_A (16.64%), Bacillota (8.15%), Actinomycetota (7.54%), Spirochaetota (5.43%), Bacteroidota (3.56%), Campylobacterota (1.56%), Cyanobacteriota (0.56%), Myxococcota (0.49%), and Patescibacteria (0.40%) (Figure 2A). The sum of the sequence proportions of these top seven phyla reached 99.15% of the total sequences. An analysis of variation was conducted on the ten most prominent phyla found across five groups, utilizing the Kruskal–Wallis test. Furthermore, the resulting *p*-values were adjusted by employing the Benjamini–Hochberg method (Figure 2B). The DJ group showed a significant increase in Campylobacterota and Spirochaetota, while the LBC group had a significant increase in Pseudomonadota and Cyanobacteriota (Figure 2B). The NR group also had a significant increase in Bacteroidota and Actinomycetota, and the RK group contained the highest amount of Bacillota_A (Figure 2B).

### 3.3. Microbial Composition and Variation in the Gut Microbiome at the Genus Level

**Figure 3:**
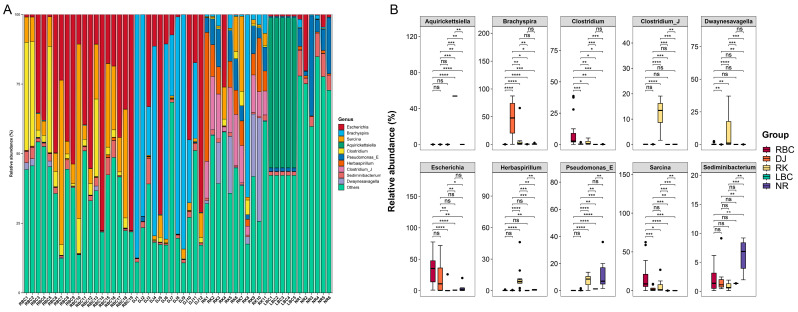
Microbial composition at genus level. (**A**) Stacked bar chart of relative abundance of the top 10 genera in each sample. (**B**) Comparison of the top 10 genera among different groups. Statistically significant differences are indicated as follows: ns, *p* > 0.05; * *p* < 0.05; ** *p* < 0.01; *** *p* < 0.001; **** *p* < 0.0001.

At the genus level, a total of 11,621 bacterial genera were annotated among the five groups (Table S3), and the top 10 genera were presented in Figure 3A. The main genera were *Escherichia* (18.13%), *Brachyspira* (12.24%), *Sarcina* (7.59%), *Aquirickettsiella* (5.09%), *Clostridium* (4.03%), *Pseudomonas_E* (3.15%), *Herbaspirillum* (2.69%), *Clostridium_J* (2.53%), *Sediminibacterium* (2.19%), and *Dwaynesavagella* (2.06%), which accounted for 59.70% of the relative abundance of all genera. The results of the differential abundance analysis showed that the RBC group had the highest levels of *Clostridium*, *Escherichia*, and *Sarcina* (Figure 3B). The DJ group had the highest content of *Brachyspira*. The RK group contained the highest amounts of *Clostridium_J*, *Dwaynesavagella*, *Herbaspirillum*, and *Pseudomonas_E*. The LBC group had the highest content of *Aquirickettsiella*. The NR group contained the highest amount of *Sediminibacterium*.

### 3.4. Analyses of Shared and Exclusive Microbial Genera

**Figure 4:**
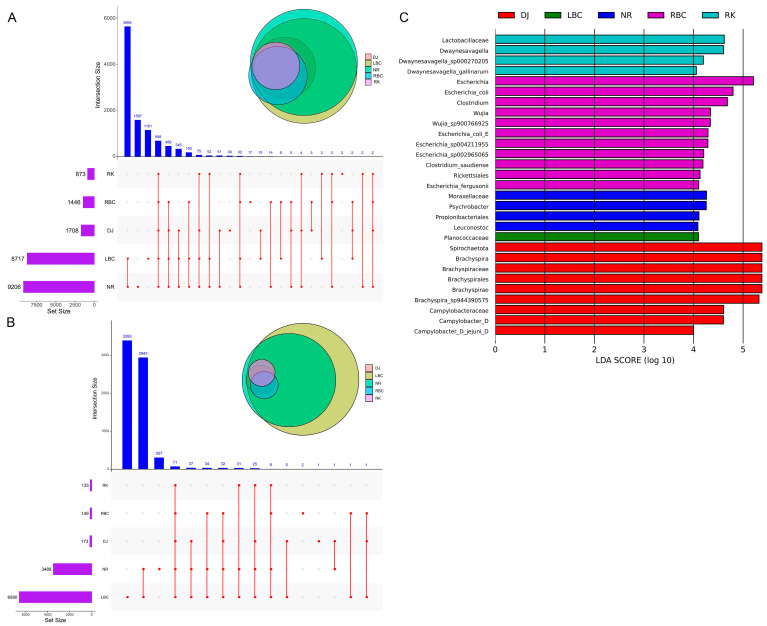
The shared and exclusive microbial genera among different groups. (**A**) The UpSet diagram shows the “core” microbial genera across all five groups, with a less stringent criterion: genera present in more than three samples within each group. (**B**) The UpSet diagram shows the “core” microbial genera across all five groups, with a more stringent criterion: genera present in 100% of samples in each group. (**C**) The LEfSe histogram displays the distribution of LDA values for the top key microbiomes that exhibit significant differences (LDAScore > 4, *p* < 0.05).

At the genus level, an analysis of shared and exclusive taxa was performed to reveal the similarities and differences in the gut microbiomes of five different crow species. Using a less stringent definition of the “core” microbial genera (genera present in more than three samples within each group), a total of 698 microbial genera were observed across all the five groups (Figure 4A), accounting for 73.62% of the total abundance of all genera (Table S4). Using a more stringent definition of the “core” microbial genera requiring that a genus was present in 100% of samples in each group, a total of 71 microbial genera were identified (Figure 4B). These core genera accounted for 53.44% of the total abundance of all genera (Table S5). Linear discriminant analysis effect size (LEfSe) was used to screen significantly different biomarkers in each group. LEfSe analysis detected a total of 259 bacterial taxonomic clades showing statistically significant differences (LDA score > 2, *p* < 0.05) among the five groups (Table S6), and the top key microbiomes in each group (LDA score > 4, *p* < 0.05) were shown in Figure 4C.

### 3.5. Alpha and Beta Diversity Analyses

**Figure 5:**
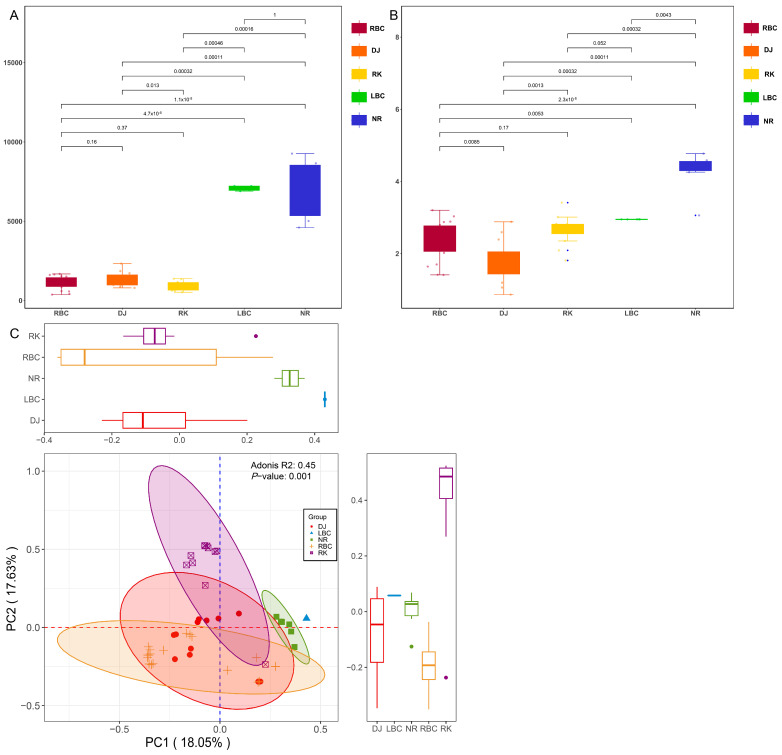
Analysis of gut microbial diversity among different groups. (**A**) The box plot of the Chao1 index. (**B**) The box plot of the Shannon index. (**C**) The principal coordinate analysis (PCoA) based on the Bray–Curtis distance indicating the significant differentiation of the bacterial species among different groups.

In order to compare the bacterial genera richness and diversity across the five groups, we analyzed the levels of bacterial richness and diversity using two specific methods. The Chao1 estimator was employed to assess bacterial richness, while the Shannon index was utilized to evaluate bacterial diversity. As illustrated in Figure 5A, the LBC and NR groups exhibited the greatest microbial richness among the samples. The Chao1 index, which serves as a measure of microbial richness, ranged from 4601.81 to 9263.37 in these groups. The RK group displayed the lowest microbial richness, with the average Chao1 index standing at 933.19. The Shannon index revealed that the NR group possessed the highest index, with an average Shannon index of 4.26, signifying the greatest bacterial diversity. Conversely, the DJ group exhibited the lowest index, with an average Shannon index of 1.73, as depicted in Figure 5B. The dissimilarity of bacterial genera among samples from five different groups was compared, and principal coordinates analysis (PCoA) based on the Bray–Curtis distance was carried out. The results showed a dispersed distribution of the microbiome across the five groups, indicating significant differences in their composition (Figure 5C).

### 3.6. Functional Annotation and Characteristics of the Crow Gut Microbial Gene Catalog

The assembly of all samples generated 3,357,068 contigs (N50 of 1482 bp; longest assembled contig is 1,055,132 bp). Summaries of assembly statistics for each sample can be found in Table S7. A total of 5,682,854 ORFs were identified in the contigs, with an average length of 450 bp (Table S8). The Venn diagram displayed the number of genes that were common and unique to each of the five sample groups (Figure S1A). PCoA was conducted to explore the relationships among the gene catalogs of the crow samples across the five groups. The results showed that the five groups clustered separately, with a variance of 45.48% accounted for by two components, PC1 and PC2, as shown in Figure S1B. After dereplication, we obtained an entire catalog of 2,738,640 non-redundant genes. The mapping of these non-redundant genes to the databases of KEGG and CAZymes revealed many enriched functional features. A total of 632,331 genes (23.09%) were annotated based on the KEGG database, and 97,946 genes (3.58%) were annotated based on the CAZymes database. These results indicated that the gut microbiomes of the five crow species contained many unknown gene functions.

**Figure 6:**
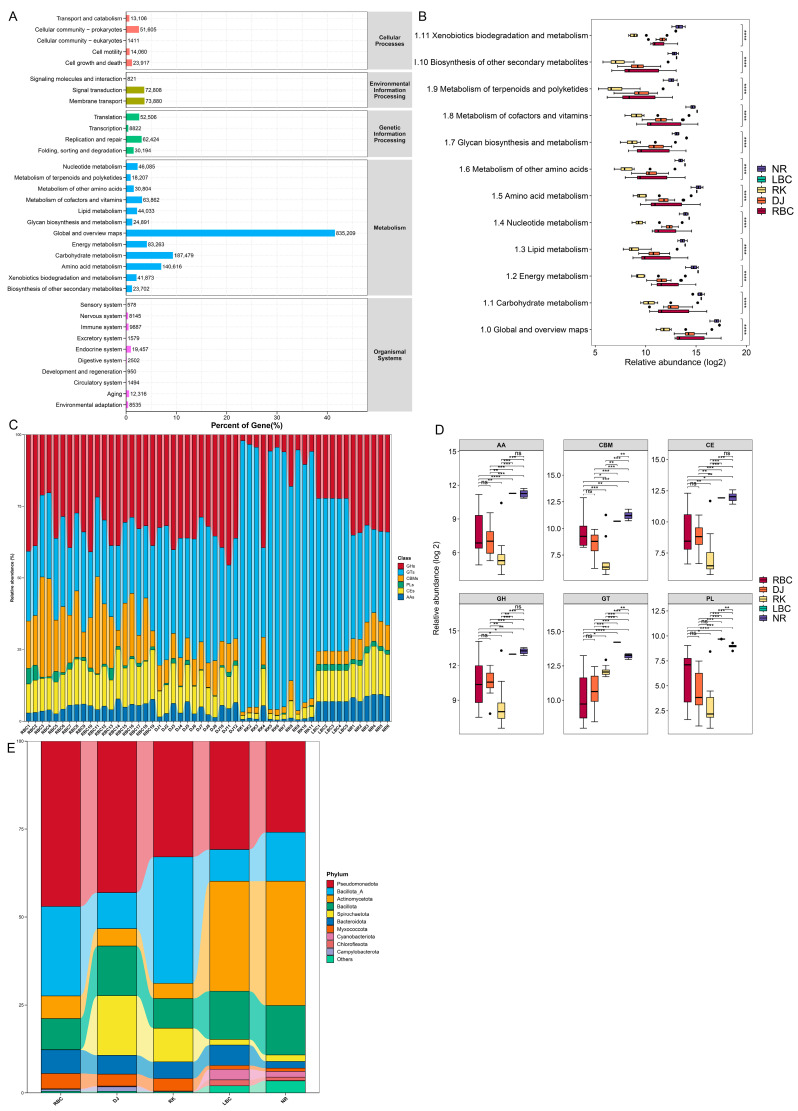
Functional prediction analysis of the gut microbiomes based on the KEGG database and the CAZymes database. (**A**) The statistical plot of KEGG annotations of the crows’ gut microbiota. (**B**) The significant functional differences in the KEGG level 2 metabolism pathways among different groups. (**C**) Stacked bar chart of relative abundance of the CAZymes classes in each sample. (**D**) Comparison of the six CAZymes classes among different groups. (**E**) The main microbial phyla contributing to CAZymes in each group. Statistically significant differences are indicated as follows: ns, *p* > 0.05; * *p* < 0.05; ** *p* < 0.01; *** *p* < 0.001; **** *p* < 0.0001.

A total of 356 KEGG pathways were annotated. The KEGG pathways were mainly enriched in metabolism (47.85%), including carbohydrate metabolism (8.63%), amino acid metabolism (6.15%), energy metabolism (5.33%), metabolism of cofactors and vitamins (5.18%), nucleotide metabolism (3.68%), and lipid metabolism (2.49%) (Figure 6A). As for the KEGG level 2 metabolism pathways, all the 12 functional terms were significantly different among the five groups (Figure 6B). Other pathways were environmental information processing (17.68%), genetic information processing (17.05%), cellular processes (11.05%), and organismal systems (6.37%).

The predicted genes were then annotated against the CAZymes database to further elucidate the mechanisms of complex carbohydrate metabolism in the gut microbiomes of the five crow species. A total of 97,946 genes were annotated to 152 types of GHs (33,929 genes), 104 types of GTs (29,572 genes), 84 types of CBMs (12,823 genes), 32 types of PLs (1581 genes), 19 types of CEs (13,165 genes), and 17 types of AAs (6876 genes), respectively. The most abundant CAZymes families among the five groups were the GTs and GHs (Figure 6C). Among the GTs, the subfamilies GT2, GT4, GT51, GT9, and GT83 were the top five major members, with relative abundances higher than those of other GTs. Among the GHs, the subfamilies GH23, GH13, GH3, GH1, and GH31 were the top five major members. Compared to the NR group, the LBC group contained higher abundances of GTs and PLs, while the NR group contained higher abundances of CBM (Figure 6D). In a comparison among the RBC, DJ, and RK groups, it was found that the RK group had the highest abundance of GTs among the six CAZymes categories, while it had the lowest abundance in the other five categories.

As shown in Figure 6E, the taxonomic classification of putative CAZyme genes in the RBC and RK groups revealed that most sequences were derived from Pseudomonadota and Bacillota_A bacteria, which collectively contributed over 68% of the genes in both groups. In the LBC and NR groups, the taxonomic classification of putative CAZyme genes showed most sequences derived from Pseudomonadota and Actinomycetota, which together comprised over 61% of the genes. In the DJ group, the taxonomic classification of the putative CAZyme genes showed that a significant portion of the sequences were derived from Pseudomonadota and Spirochaetota bacteria. These two bacterial groups contributed over 59% of the genes in the DJ group, highlighting their predominance in terms of potential CAZyme gene contributions.

### 3.7. Profiles of VFGs and Bacterial Pathogens

**Figure 7:**
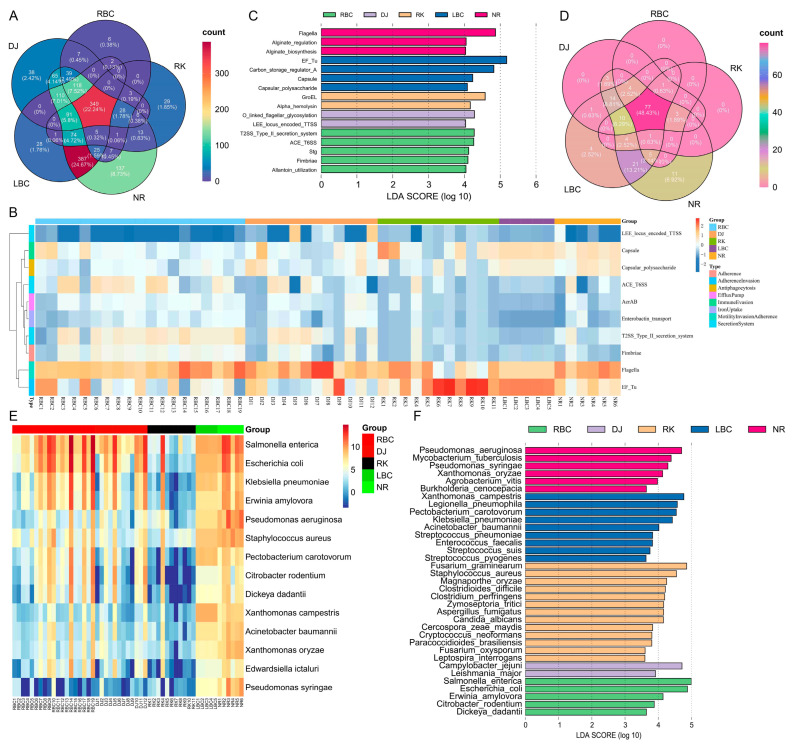
Profiles of VFGs and bacterial pathogens in the crows’ gut microbiota. (**A**) The Venn diagram shows the number of shared VFGs among different groups. (**B**) The heatmap of the top ten virulence factors. (**C**) The LEfSe histogram displays the distribution of LDA values for the key VFGs subtypes that exhibit significant differences (LDAScore > 4, *p* < 0.05). (**D**) The Venn diagram shows the number of shared pathogenic bacteria among different groups. (**E**) The heatmap of the top 14 pathogenic bacteria. (**F**) The LEfSe histogram displays the distribution of LDA values for the key pathogenic bacteria that exhibit significant differences (LDAScore > 3, *p* < 0.05).

Bacterial infections typically involved virulence factors, which were vital for bacterial pathogenicity. The identification of these virulence factor genes (VFGs) was key to the effective treatment and management of severe bacterial infections. In this study, a total of 3607 genes (0.13%) were annotated based on the virulence factor database. The NR group had the highest number of VFGs (2551), followed by the DJ group (463), the LBC group (437), the RBC group (85), and the RK group (71). In this study, 22.24% of the detected VFGs were shared among the five groups (Figure 7A). A total of 43 types of VFGs with 243 subtypes were identified across all samples. Among the top ten virulence factors, the enrichment of VFGs was mainly in offensive VFGs, which was related to the function of motility, secretion systems, and adherence (Figure 7B). The defensive VFGs involved immune evasion, anti-phagocytosis, efflux pump, and iron uptake. The LEfSe analysis indicated that a total of 16 VFGs subtypes (LDA score > 4, *p* < 0.05) were the key markers among the five groups (Figure 7C).

To determine the presence and abundance of pathogenic bacteria in the gut of the five crow species, a total of 22,889 genes (0.84%) were annotated against the Pathogen–Host Interactions Database. A total of 159 pathogens were detected, involving 284 types of diseases related to 95 hosts. A total of 77 pathogens (48.43%) were shared among the five groups (Figure 7D). Five “*ESKAPE*” pathogens (*Enterococcus faecium*, *Staphylococcus aureus*, *Klebsiella pneumonia*, *Acinetobacter baumannii*, and *Pseudomonas aeruginosa*) were observed in the shared pathogens. As shown in Figure 7E, the total abundance of the top 14 pathogenic bacteria accounted for 86.76% of all the data, representing the main pathogens in this study. LEfSe analysis indicated that a total of 35 pathogens (LDA score > 3, *p* < 0.05) were the key markers among the five groups (Figure 7F). Further statistical analysis of 95 host species revealed that the proportion of hosts related to vertebrates was 26.32% (25 species), that related to invertebrates was 20% (19 species), and that related to plants was 53.68% (51 species), suggesting that these crow species may share pathogen transmission pathways with these hosts.

### 3.8. Metagenome-Assembled Genomes (MAGs) from the Crow Gut Metagenome

**Figure 8:**
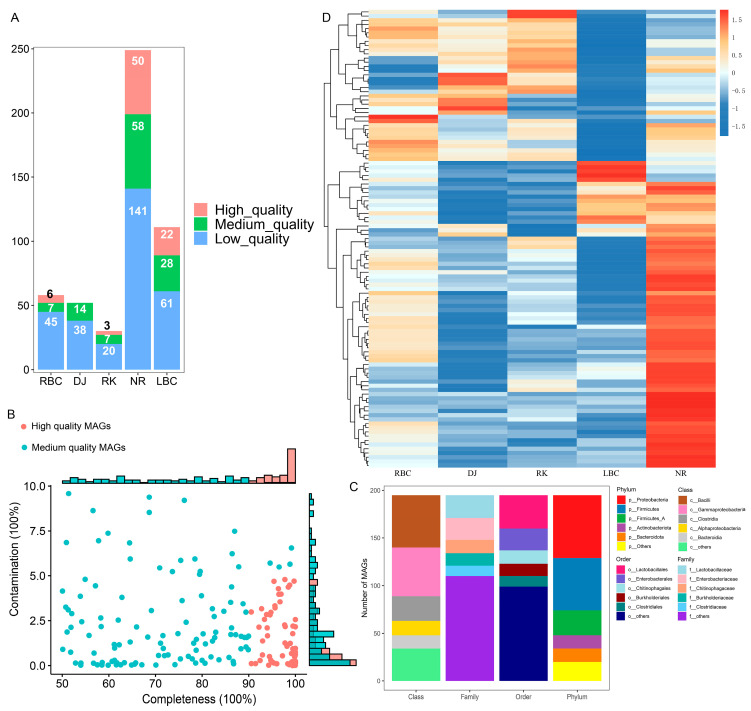
(**A**) The number of high, medium, and low-quality MAGs in each group. (**B**) Estimated completeness and contamination of 500 MAGs recovered from crows’ gut metagenomes. Medium-quality genomes are shown in green, and high-quality genomes in red. Histograms along the x and y axes showing the percentage of genomes at varying levels of completeness and contamination, respectively. (**C**) Taxonomic distribution of the MAGs at phylum, class, order, and family levels. Only the top five taxa were shown at each taxonomic level, and the remaining ones were grouped as “others”. (**D**) Heat map showing the significant differences in MAGs among different groups.

We employed a single-sample assembly approach to reconstruct the microbial genomes from all 53 metagenomic datasets. A total of 500 MAGs were obtained (Figure 8A). The NR group and the LBC group obtained the highest number of MAGs (Figure 8A). Within these groups, 81 MAGs exhibited a completeness rate exceeding 90% (classified as high-quality), which included 18 MAGs that demonstrated a completeness of 100%. A total of 114 MAGs displayed a completeness ranging from 50% to 90% (categorized as medium-quality), while the remaining 305 MAGs exhibited a completeness of less than 50% (considered as low-quality) (Figure 8B). The genome taxonomy database toolkit (GTDB-Tk) was then used to conduct taxonomic assignments of the 195 high-quality and medium-quality MAGs. Of these MAGs, 123 (63.08%) were identified as matches to known microbial species, indicating that around 36.92% of the MAGs (72 in total) could originate from unknown species not yet represented in the existing database and may signify new species (Table S9). Overall, the 123 MAGs were taxonomically assigned to 10 phyla, 13 classes, 30 orders, and 44 families, spanning across 69 genera. Most of them belonged to Proteobacteria (34.74%), followed by Firmicutes (28.95%), Firmicutes_A (13.68%), Actinobacteriota (7.37%), and Bacteroidota (7.37%) (Figure 8C). The number of predicted genes in 109 nonredundant MAGs ranged from 402 to 8186, with an average length between 726 bp and 1176 bp, summing up to a total of 300,821 genes. We then quantified and compared abundances of the 109 nonredundant MAGs in the metagenomes data. The abundances of 55 MAGs across five groups exhibited significant differences (Kruskal–Wallis test, *p* < 0.05), and the differential MAGs were visualized using a heatmap (Figure 8D). A comprehensive list of 1005 human pathogen species names was compiled from the literature [37], and by matching the MAGs annotated to the species level with this list, a total of 33 MAGs were identified as potential pathogens (Table S10). These 33 MAGs belonged to three phyla, eight genera, and eight species in terms of taxonomic classification. Among them, the phylum with the largest number of MAGs (*n* = 22) was Proteobacteria, and the species with the largest number of MAGs (*n* = 18) was *Escherichia coli*. A total of 231 VFGs were identified across all 18 *Escherichia coli* MAGs. In detail, the VFG analysis of these *Escherichia coli* MAGs showed that adherence- and motility-related genes accounted for 48.76%, suggesting a dominant offensive types. This indicates strong colonization and fast spread capabilities, which is crucial for pathogenicity.

## 4. Discussion

In this study, we characterized the gut microbiomes of five typical crow species, with a total of 53 samples from the Qinghai–Tibetan Plateau. Using a metagenomic approach, we generated a comprehensive gene catalog of crow gut microbiomes consisting of 2,738,640 unique genes and reconstructed 195 microbial genomes. This significantly enhanced the existing repository of high-quality MAGs from wild bird guts and represented the most extensive collection of genes and genomes for crows to date. The extensive gene catalog offers a vital foundation for exploring the crow gut microbiota and notably simplifies the analysis of metatranscriptomes and metaproteomes using mapping techniques in future studies. Additionally, the MAGs reported here serve as a valuable genomic resource, significantly expanding the catalog of uncultured microbial reference genomes.

The microbial community in the avian gut was primarily dominated by Firmicutes, with lesser contributions from phyla including Actinobacteria, Bacteroidetes, and Proteobacteria [11]. This study revealed that Pseudomonadota had a relative abundance, exceeding 50% in the gut microbiota of crows at the phylum level. The elevated presence of Pseudomonadota might be associated with the cohabitation of crows and humans, as investigations revealed that the gut microbiota of urban house sparrows (*Passer domesticus*) contained a greater number of microorganisms from the phylum Pseudomonadota, which played a significant role in various mammalian intestinal and extraintestinal diseases [38]. This also indirectly indicated that a bird’s exposure to humans could substantially impact the microbes present in avian intestines [39]. Additionally, the relative abundances of Bacillota_A, Bacillota, Actinomycetota, Spirochaetota, and Bacteroidota were found to be moderate in the gut microbiota of crows. These phyla were frequently observed in the gut microbiota of birds, suggesting that the avian gut core microbiota constituted a relatively stable group of microbial communities. This stability likely facilitated better adaptation to natural conditions and might have crucial functional roles in gastrointestinal tracts, such as digestion, nutrient absorption, and immune responses [40]. As corvids are omnivorous birds, we speculated that these microbial communities were related to the digestion and utilization of food by crows. For example, Pseudomonadota are a diverse group of bacteria that are known for their metabolic versatility. This ability to grow on a range of organic compounds, including proteins, carbohydrates, and lipids, makes them important inhabitants of various environments, such as soil, water, and the guts of animals, including humans [41]. Bacillota_A and Bacillota played key roles in the digestion and energy metabolism of fiber in the hosts they inhabited. Their abundance had been linked to obesity in humans [42] and weight gain in chickens [43], indicating a possible role in energy storage. Despite the lack of studies on wild birds, the positive relationships observed in domestic chickens hinted at potentially conserved roles across different species. The phylum Bacteroidota was widely recognized for its importance in the degradation of complex polysaccharides, including cellulose and other plant fibers, in the digestive systems of herbivorous animals, including birds [44]. Birds have a more efficient digestive system compared to mammals, which is reflected in their shorter intestinal tracts and shorter intestinal content retention times. The connection between these microbial communities and the efficient digestion of crows deserves further research in the future. Another key influence on the development of gut microbiota involved alterations in the oxygen levels within the host’s intestinal cavity. The intestinal environment maintained an ecological pattern characterized by a prevalence of anaerobic bacteria and a scarcity of aerobic bacteria. Birds were found to have fewer obligate anaerobes and more facultative anaerobes compared to mammals [10]. Since Pseudomonadota was the most abundant phylum in the crow gut microbiome, we hypothesized that these bacteria play a role in maintaining the homeostasis of the anaerobic environment of the gastrointestinal tracts, and hence, the stability of the strictly and facultative anaerobes. Variations in the relative abundances of the dominated phyla among crow species were also detected in our study. These differences might be due to variations in species, diets, living environment. Comprehending the interplay between diverse host and environmental factors in shaping the gut microbiota has been a key objective in modern bird microbial ecology and evolutionary studies [45]. Nevertheless, due to the absence of adequately precise comparisons in our research, we were unable to identify the underlying causes of the disparities in the intestinal microbiota among different crow species.

At the genus level, we found that the crows’ gut microbiota contained many pathogenic or opportunistic pathogenic microorganisms, such as *Escherichia* [46], *Brachyspira* [47], *Sarcina* [48], *Clostridium* [49], which were associated with diseases in multiple species. We speculated that crows, due to their broad diet and strong adaptability, are widely distributed across various habitats, which is why they carry so many intestinal pathogens. Pathogens could play a role in the decline in wild bird populations by increasing mortality and reducing reproductive success. Additionally, some infectious agents in wild birds possess zoonotic potential and are relevant in the context of poultry. How the gut microbiome of these pathogens influences the health status of the crows remains unknown and requires further investigation in the future. Multiple studies have examined the intestinal pathogens of wild birds through cultural methods, focusing primarily on specific pathogens [50,51]. However, as these culture-based investigations offered only a restricted insight into the natural microbial pathogenic communities, it became crucial to use culture-independent approaches, such as the metagenomic method used in this study, to accurately determine the composition of the pathogens in the gut microbiome. Furthermore, based on the Pathogen–Host Interactions Database, at the species level, we identified a total of 159 pathogens, involving 284 types of diseases, across 95 host species. These data aided us in further understanding the pathogens carried by crows. Nearly half of the pathogens were found to be common among five species of crows, indicating that these birds may share similar habitats, behaviors, or transmission pathways, which contribute to the spread of these microorganisms. Among the pathogens common to the five species of crows, we also, surprisingly, found five “*ESKAPE*” pathogens. The *ESKAPE* pathogens, which have developed resistance to nearly all antibiotics used against them, pose a primary threat due to their ongoing worldwide spread of drug resistance [52]. In the future, it will be necessary to further examine the multidrug resistance of these *ESKAPE* pathogens using bacterial isolation and culture methods. These findings highlight the necessity for additional studies to comprehend the epidemiology of these pathogens, their persistence in the environment, and their effects on crow populations, as well as their potential transmission to other species, including humans. Before we could use crow species as indicators of environmental changes and potential threats from pathogens, it was essential to establish a basic understanding of the composition of the microbiomes in wild crows. This was also the significance behind conducting our study.

The “core microbiome” was considered a vital component of the fundamental functions of holobionts, which were enriched, selected, and inherited through evolutionary processes [53]. Using different criteria, we obtained the core microbiome of five species of crows, and even 71 genera were found to exist in all 53 samples. These bacteria were likely indispensable for the host to adapt to extreme diets or environments. The results of our core gut microbiota were derived from a single time point in a large cohort of hosts. In reality, the core gut microbiota is not necessarily confined to a certain point in time; it can be defined on a larger temporal scale throughout the lifespan of the host, such as a core composed of microbes that remain stable throughout the entire life of the host [54]. Future studies could focus on excavating the core gut microbiota across the full life cycle of specific crow species. Correspondingly, we also analyzed significantly different biomarkers in the gut microbiota of five species of crows, involving 259 bacterial taxonomic clades. The microbial composition was primarily influenced by various factors, including intrinsic factors such as host genetics and the immune system, and extrinsic factors such as diet and the environment [55]. Thus, to understand what caused the distinct gut microbiota in each of the five crow species, we needed to disentangle the relative importance of these factors in the future. Based on the sampling locations, the Red-billed chough group and the Daurian jackdaw group were sampled at closer proximity, while the remaining three groups (the Rook group, the Large-billed crow group, and the Northern raven group) were sampled further apart. Correspondingly, the beta diversity results of the gut microbiota indicated that there was a higher overlap between the Red-billed chough group and the Daurian jackdaw group, while the other three groups were distinctly separate. All five species of crows were resident birds and did not engage in long-distance migration. Besides geographical location, we speculated that diet, which is related to geographic factors, was another determining factor in the differences in gut microbiota among the five crow species. Nevertheless, the majority of research on the gut microbiomes in wild birds has depended on the literature to categorize bird species into specific dietary groups, such as insectivores or frugivores [2]. In this study, we only knew that crows are omnivorous, with insects, plants, carrion, and more being part of their diet, but it was unclear about the specific differences in the dietary composition among the five crow species. From the results of gut microbiota alpha diversity, we found that the Northern ravens had the highest Chao1 and Shannon indices. The reason could be that Northern ravens, with their large size, strong adaptability, and wide ecological niche, have access to a rich variety of food sources [56]. Additionally, known for their high intelligence, ravens’ social interactions might facilitate the transmission of microbes between individuals, thereby increasing the likelihood of diverse microbial communities [57]. In our results, the Rooks had the lowest Chao1 index, and the Daurian jackdaws had the lowest Shannon index. This is an interesting direction for future exploration, investigating whether the smaller size, more specialized food sources, or weaker social behaviors of these two crow species led to lower richness and diversity of gut microbiota. Crows also have the potential to become an excellent model species for studying the interaction between intelligence and gut microbiota.

The present study also analyzed the function of the crows gut microbiota based on the KEGG, CAZymes, and VFDB databases. The analysis revealed that the primary functions of the microbiota in crows were heavily involved in metabolism-related pathways, especially carbohydrate metabolism. Other major pathways involved were amino acid metabolism, energy metabolism, metabolism of cofactors and vitamins, nucleotide metabolism, and lipid metabolism, etc. These metabolic functions also had species differences and might have correlated with distinct bacteria phyla and genera. Interestingly, variations in carbohydrate metabolism enzymes were also observed among crow species. These differences indicate a differential metabolism of carbohydrates, likely reflecting a more diverse diet, with more complex and variable carbohydrate availability. Birds’ utilization of dietary fiber is inefficient due to the lack of enzymes capable of digesting fiber [58]. Therefore, the microbiota in the guts of birds that consumed plant-derived fiber may have compensated for the absence of fiber-digesting enzymes in the host. The most abundant CAZymes families were GTs and GHs among the five groups of crows, which were the key enzyme families involved in the assembly (GTs) and breakdown (GHs) of carbohydrate complexes [59]. For example, GHs are enzymes that break down glycosidic bonds in complex carbohydrates such as cellulose, hemicellulose, and starch. In crows, the GH families that degrade hemicellulose compounds, specifically GH1, GH3, and GH31, composed the majority of all the GH genes. This indicates that the bacteria in the crow’s gut had a strong ability to break down these types of polysaccharides. Another abundant GH family in crows, GH13, was found to be directly proportional to the digestibility of animals [60]. Such diversity of GHs families could have allowed crows to readily adapt to the plant components in their omnivorous diet. Upon further analysis of the species classifications related to these CAZyme genes, it was discovered that these genes were almost exclusively associated with the phylum Pseudomonadota, which was consistent with the observed high abundance of Pseudomonadota. Besides the bacteria from Pseudomonadota, different crow species also had distinct gut microbiomes that contributed CAZyme genes, which could potentially explain the variations in the CAZyme genes observed among different crows. Theoretically, each gut microbial community, due to the specificity of its genome, was capable of degrading specific complex carbohydrates. As a result, alterations in the composition of the microbiota would result in differences in the ability of the microbial community to digest complex carbohydrates. This also suggests that in the future, linking the dietary composition of birds with the composition and function of their gut microbiota would help to better understand the function of the gut microbiome. Our study also discovered a series of VFs in the crows, suggesting that these birds harbor bacteria that could be potentially harmful. This finding raises concerns not only about the native survival of these birds but also possible health risks to humans. Crows, in particular, carry a multitude of pathogens, as we detected, and the pathogens harboring VFGs could cause diseases and facilitate the exchange of VFGs among bacterial pathogens across various environments [61].

The reconstruction of microbial genomes from metagenomic sequences has substantially increased the number and diversity of microbial genomes, especially for those strains that cannot be cultivated [13]. Therefore, in this study, we reconstructed a total of 195 high-quality and medium-quality MAGs from 53 crows fecal metagenomes. The taxonomic categorization of the reconstructed MAGs matched the predominant microbial composition in the crows’ gut, as shown by the results of the metagenomic assembly. This also suggested that the genomes of microbes with higher abundance were recovered at a higher rate. Likewise, 33 pathogen-related MAGs were discovered, primarily associated with *Escherichia coli*. The key genes associated with motility and adhesion in these *Escherichia coli* MAGs were also detected, indicating that these bacteria might possess the ability to actively move and adhere to host cells, which are crucial factors in pathogenicity and colonization. Therefore, monitoring and control efforts should have focused on *Escherichia* species, particularly *Escherichia coli*, which was considered a significant indicator of pathogens. While metagenomic studies can aid in the assembly of microbial genomes, traditional culturing followed by complete genome sequencing remains the most reliable method. To fully comprehend the diverse bacterial populations in crows and their roles, it was essential to employ both cultivation techniques and sequencing approaches.

In the gut microbiome study of the five crow species in this research, fecal samples were collected from two species, and intestinal content samples were collected from the remaining three species (due to the unavailability of fecal samples). Overall, as two commonly used sampling methods for avian microbiome studies, both the fecal approach and the intestinal content approach had their strengths and weaknesses. For example, fecal collection was a convenient, non-invasive method for examining the gut microbiome but risked contamination and time delays. In contrast, intestinal content sampling provided the immediate preservation of the microbiome from euthanized animals, bypassing community shifts in fecal samples. However, this method was not suitable for repeated sampling of individuals or large-scale studies due to conservation concerns and permitting constraints. A key question in comparing fecal and intestinal sampling methods was how different the gut bacterial communities from these sample types were, but this was not examined in our study. For these five crow species, we could not quantify the extent of differences in gut microbiota resulting from different sampling methods, as no analytical methods existed to minimize potential compositional differences. To address this question in a wild bird system, in the future, it will be necessary to compare and analyze fecal samples and intestinal content samples obtained from the same individuals of the same species.

## 5. Conclusions

In summary, this was the first study to evaluate the gut microbiome of five typical corvid bird species from the Qinghai–Tibetan Plateau. The results clearly demonstrated differences in the composition, diversity, and functional structures of the gut microbiota among the five crow species. The first gene sets and the constructed microbial genomes of the crows’ gut microbiome developed in the current study offered valuable resources for gaining insights into the gut microbiome of corvid birds. They also provide a foundation for future metagenomic sequencing-based studies. The results from our study warrant a detailed investigation of the pathogen profiles in crows’ gut microbiomes to assess the actual extent of this overarching threat to human health.

## Data Availability

The raw sequence data from this paper have been deposited in the Genome Sequence Archive at the National Genomics Data Center, China National Center for Bioinformation/Beijing Institute of Genomics, Chinese Academy of Sciences (GSA: CRA018740, CRA018741, CRA018833, CRA018712, CRA018950), accessible at https://ngdc.cncb.ac.cn/gsa (accessed on 1 September 2024).

## Supplementary Materials

Supplementary data associated with this article can be found in the online version at: https://www.mdpi.com/article/10.3390/microorganisms12102033/s1, Figure S1: Profiles of functional genes in the crows’ gut microbiota. (A) Venn diagram showing the number of shared predicted genes among different groups. (B) The principal coordinate analysis (PCoA) based on the Bray–Curtis distance indicating the significant differentiation of the functional genes among different groups; Table S1: Summary of the 53 metagenomic data, including raw sequencing data, quality-controlled clean data, and data subsequent to host removal for each sample; Table S2: Abundance information at the phylum level for each sample; Table S3: Abundance information at the genus level for each sample; Table S4: Abundance information of 698 core gut bacterial genera in each sample; Table S5: Abundance information of 71 core gut bacterial genera in each sample; Table S6: Results of the LEfSe analysis demonstrating a total of 259 bacterial taxonomic clades that exhibited statistically significant differences (LDA score > 2, *p* < 0.05) among the five groups; Table S7: Summaries of assembly statistics for each sample; Table S8: Summaries of ORFs for each sample; Table S9: Taxonomic assignments of the 195 high-quality and medium-quality MAGs; Table S10: Pathogenic classification information for 33 MAGs.
