# Supplementary material for: The Catalog of Microbial Genes and Metagenome-Assembled Genomes from the Gut Microbiomes of Five Typical Crow Species on the Qinghai–Tibetan Plateau
Source: Microorganisms. 2024 Oct 8;12(10):2033. doi: 10.3390/microorganisms12102033 (PMC11510465; doi:10.3390/microorganisms12102033)
Supplement: Supplementary file 1 [file microorganisms-12-02033-s001.zip › Supplementary_Material_Figure S1.docx]

**Supplementary Material**


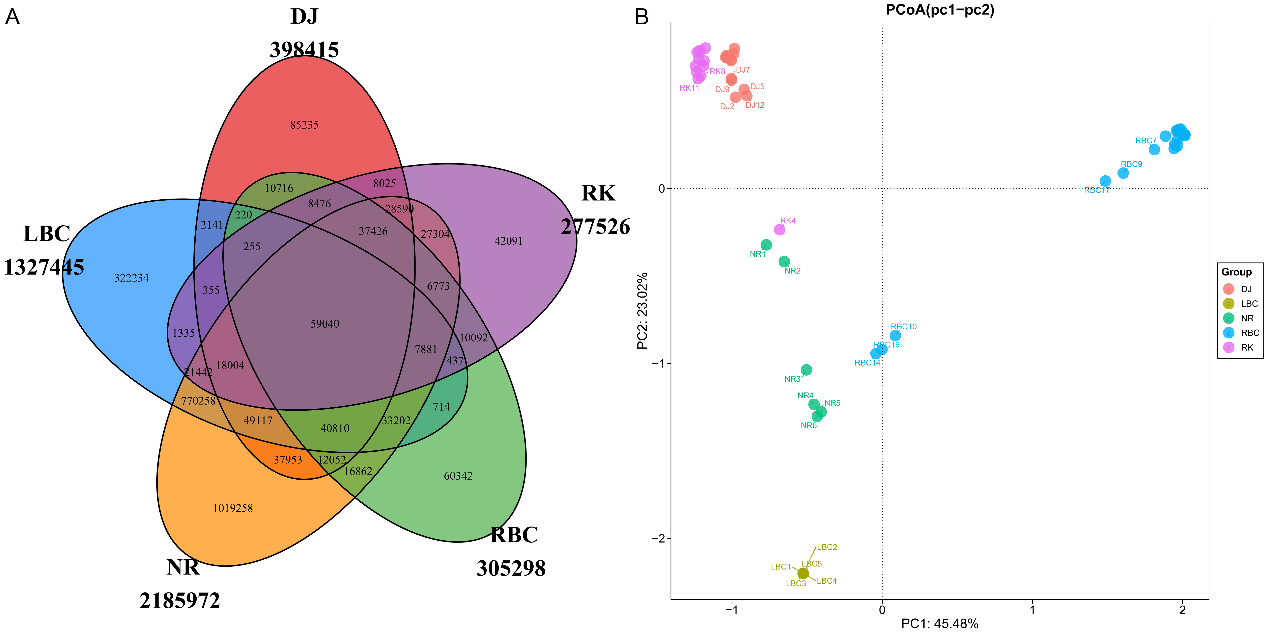


**Figure S1. Profiles of functional genes in the crows’ gut microbiota.** **(A)** Venn diagram showed the number of shared predicted genes among different groups. **(B)** The principal coordinate analysis (PCoA) based on the Bray-Curtis distance indicating the significant differentiation of the functional genes among different groups.
